# Myofascial trigger points in patients with temporomandibular joint disc displacement with reduction: a cross-sectional study

**DOI:** 10.1590/1678-7757-2017-0578

**Published:** 2018-05-29

**Authors:** Rodrigo Lorenzi POLUHA, Eduardo GROSSMANN, Lilian Cristina Vessoni IWAKI, Taqueco Teruya UCHIMURA, Rosângela Getirana SANTANA, Liogi IWAKI

**Affiliations:** 1 Universidade Estadual de Maringá, Departamento de Odontologia, Maringá, Paraná, Brasil.; 2 Universidade Federal do Rio Grande do Sul, Faculdade de Odontologia, Porto Alegre, Rio Grande do Sul, Brasil.; 3 Universidade Estadual de Maringá, Departamento de Estatística, Maringá, Paraná, Brasil.

**Keywords:** Trigger points, Temporomandibular joint, TMJ disorders, Facial pain

## Abstract

**Objective:**

The objective of this retrospective study was to evaluate the impact of myofascial trigger points (MTrPs) in patients with articular disc displacement with reduction (DDWR) and to identify which clinical variables are associated with the concomitant presence of DDWR and MTrPs.

**Material and Methods:**

130 patients were selected that sought treatment due to joint pain, with ages ≥18 years, of both genders, with DDWR confirmed by magnetic resonance imaging. The sample was divided into two groups: Group 1, patients with DDWR and MTrPs (N=101); and Group 2, patients with DDWR and no MTrPs (N=29). Information on gender, age, pain duration, pain scores, and maximal interincisal distance (MID) were collected. The logistic regression model was used and the odds ratios (OR) was calculated (p<0.05).

**Results:**

Group 1 presented statistically significant higher mean pain scores (p=0.007), and smaller MID (p=0.0268) than Group 2. OR were significant for the pain scores (1.429), MID (0.937) and gender (women) (2.810).

**Conclusions:**

Patients with DDWR and MTrPs had increased pain scores and a MID decrease compared to patients with DDWR and no MTrPs. The variables pain scores, MID, and gender (women) showed a significant association with the concomitant presence of DDWR and MTrPs.

## Introduction

Temporomandibular disorders (TMDs) are a group of musculoskeletal disorders that affect the stomatognathic system^1^. TMD-related pain can negatively impact normal daily activities and the psychosocial functioning of an individual^2^. Besides that, there is a significant reduction of life quality of patients with specific subtypes of TMDs, such as articular disc displacement with reduction (DDWR)^3^ and myofascial pain (MFP)^4^. Patients with two TMD-related pain diagnoses have more impairment of life quality than subjects with one diagnosis^4^.

DDWR is one of the most common internal derangements of the TMJ^5^. In patients with DDWR, when the mouth is closed, the articular disc is anteriorly displaced in relation to the condyle and, when the mouth is open, the disc returns to its original position, in the intermediate area between the condyle and the articular tubercle^1^ ^,^ ^6^ ^,^ ^7^. Although most cases of DDWR are not painful, pain, when present, is due to TMJ arthralgia^7^. Arthralgia is the TMJ inflammation, generating pain and sensitivity^1^ ^,^ ^7^. In isolation, DDWR responds for about 30% of all TMDs, increasing to about 50% when combined with muscle conditions^8^ ^,^ ^9^.

MFP alone represents 45.3% of TMD diagnoses^10^ and is defined as a regional muscle pain associated with tenderness on palpation and referred pain^1^. Clinically, referred muscle pain is linked to myofascial trigger points (MTrPs)^11^. MTrPs are hypersensitive points located in a taut band of a skeletal muscle, which can cause specific local or referred pain on stimulation and follows a reproducible pattern^12^ ^-^ ^14^. From a clinical perspective, MTrPs can be differentiated by manual assessment into active and latent^12^. MTrPs are classified as active when they cause spontaneous pain and latent when they only provoke pain when stimulated^12^. MTrPs can be result of several factors such as trauma, hypovitaminosis, fatigue and even viral infections^12^ ^,^ ^13^.

The literature shows an important relation between MTrPs and several conditions such as fibromyalgia, migraine, chronic tension-type headache and osteoarthritis^11^ ^,^ ^14^. However, MTrPs still have not been addressed in relation to patients with DDWR. This knowledge can contribute to improved understanding of these conditions and also help in their clinical decision process. Therefore, the objective of this retrospective study was to evaluate the impact of MTrPs in patients with DDWR, and to identify which clinical variables are associated with the concomitant presence of DDWR and MTrPs. The null hypothesis to be tested is that the studied variables would not present differences between patients with or without MTrPs and none of them would be associated with the presence of MTrPs in individuals with DDWR.

## Material and methods

This retrospective observational cross-sectional analytical study was approved by the Ethics Committee for the Research Involving Human Beings of the State University of Maringá, Maringá, Brazil (Number: 1.664.590/2016). This study was conducted in accordance with the recommendations of the Strengthening the Reporting of Observational Studies in Epidemiology (Strobe) guidelines^15^ and in conformance with the Helsinki Declaration. All the data used in this study were secondary, and were collected from the clinical records of patients treated for TMD at the Orofacial Pain and Deformity Center (CENDDOR), Porto Alegre, Brazil, between January 2005 and May 2016. All clinical examinations and data collection were conducted by a single examiner, specialist in TMJ disorders.

For this study, initial selection encompassed records of 520 individuals that sought treatment due to joint pain, with ages ≥18 years, of both genders, with clinical signs and symptoms of intra-articular TMJ disorders compatible with DDWR, according to the parameters and criteria established by the Research Diagnostic Criteria for Temporomandibular Disorders (RDC/TMD) - Axis I^1^, in the official Portuguese version. DDWR diagnostics were confirmed by magnetic resonance imaging (MRI). All MRI examinations were performed with a 1.5-T imaging system (Signa HDxt; GE Healthcare, Milwaukee, WI, USA) and analysed by the same experienced radiologist, according to the criteria defined by Ahmad, et al.^6^ (2009). Patients were excluded from the study when presenting no joint pain, with any other TMD conditions (except DDWR, MFP and arthralgia), with rheumatoid arthritis, agenesis, hyperplasia, hypoplasia and/or malignant neoplasm of the condyle, bone ankylosis, with neurological diseases, primary headaches, fibromyalgia, removable dental prostheses, or were making the continuous use of medications such as analgesics, benzodiazepines, antipsychotics or antidepressants. Patients who had any previous treatment for their TMD conditions, or had any previous surgical intervention in the TMJ and/or neck and head, were also excluded from the study. A total of 130 patients fulfilled the study criteria.

Information about the presence, quantity, location and classification of the MTrPs were also obtained from the records. MTrPs data were obtained during bilateral muscle palpation of the anterior, middle and posterior temporalis; masseter, upper trapezius and sternocleidomastoid by an examiner with more than 30 years of experience in MTrPs examination. MTrPs examination in these muscles was performed following the criteria described by Gerwin, et al.^16^ (1997) and by Simons, et al.^12^ (1999): 1) presence of a palpable taut band in a skeletal muscle; 2) presence of a hyperirritable tender spot within the taut band; 3) local twitch response elicited by the snapping palpation of the taut band; and 4) presence of referred pain in response to MTrPs compression (approximately 20-N force for 5 seconds). MTrPs were considered active when they caused spontaneous pain, and latent when pain was provoked only when stimulated^12^.

The following clinical data were studied: gender; age (years); pain duration (months); pain scores (0-10) obtained with the visual analogue scale (VAS)^17^ ^,^ ^18^ about the patient’s complaint; and maximal interincisal distance (MID)^18^ in millimetres (mm), which was obtained by requesting patients to slowly and steadily open their mouths, even when painful. The distance between the incisal edge of the central upper and lower incisors adding the overbite was measured. This measuring was done with the aid of a digital caliper (Mitutoyo, Takatsu-ku, Kawasaki, Kanagawa, Japan).

To evaluate the impact of MTrPs in patients with DDRW, the sample was divided into two groups: Group 1 (n=101): patients with DDWR and MTrPs; and Group 2 (n=29): patients with DDWR and no MTrPs. All demographic data of the groups, frequency distribution, means and standard deviations (SD±) of the variables studied are presented in Table 1.

**Table 1:** Demographic data of the groups, frequency distribution, means and standard deviations (SD±) of the variables studied

VARIABLES		Group 1 (n=101)	Group 2 (n=29)	Total (n=130)
Gender	Female	77 (76.23%)	15 (51.72%)	92 (70.76%)
	Male	24 (23.77%)	14 (48.28%)	38 (29.24%)
Age (Years)		36.42±13.49	35.72±13.85	36.45±14.06
Pain Duration (Months)		33.36±47.69	25.41±44.45	31.58±46.94
VAS Pain Scores (0-10)		5.97±1.84	4.89±1.58	5.73±1.84
Maximal Interincisal Distance (mm)		41.54±8.38	45.11±7.41	42.34±8.28

Group 1: patients with DDWR and MTrPs; Group 2: patients with DDWR and no MTrPs

### Statistical analysis

All data were tabulated and submitted to a descriptive analysis. The Hosmer and Lemeshow logistic regression test^19^ was applied to compare the groups. Adopting the individual as the observational unit, gross odds ratio (OR) was calculated by univariate analysis. To better understand the relative contribution of each variable to the dependent variables (the concomitant existence of DDWR and MTrPs or only DDWR and no MTrPs), variables that demonstrated a p-value ≤0.05 were subjected to multivariate analysis with the multiple logistic regression model for adjusted OR calculation. Then, the goodness-of-fit test for the variables composing the multiple logistic regression model was assessed.

All the tests were conducted with SAS version 9.3 (SAS Institute Inc., Cary, NC, USA) with a 5% significance level.

## Results

In the gross OR calculation performed with univariate analysis comparing Groups 1 and 2, only the variables VAS pain scores, MID, and gender (women) demonstrated to be statistically significant (p-value <0.05). Then, these three variables were analyzed through multivariate analysis. The variables age (p=0.8048) and pain duration (p=0.4254) showed no statistical significance, and were therefore excluded from additional analyses (Table 2).

**Table 2:** Univariate analysis – Gross odd ratios (OR)

Variables	p-value	Gross OR	95% Confidence Limits
Age	0.8048	1.004	0.973 – 1.036
Pain Duration	0.4254	1.004	0.994 – 1.015
VAS Pain Scores	0.0069*	1.390	1.095 – 1.765
Maximal Interincisal Distance	0.0431*	0.946	0.896 – 0.998
Gender (women)	0.0125*	2.994	1.267 – 7.080

* Statistically significant

Multivariate analysis was performed with the multiple logistic regression model. The variables VAS pain scores, MID, and gender (women) were analysed together. The results showed that the three variables were, in fact, significantly associated with the concomitant presence of DDWR and MTrPs. Comparing the groups, patients in Group 1 presented statistically significant higher mean VAS pain scores and smaller MID than patients in Group 2 (p<0.05) (Table 3). The goodness-of-fit test indicated that the model and the variables used were significantly well fitted (p=0.5962).

**Table 3:** Multivariate analysis - Adjusted odd ratios (OR)

Variables	p-value	Adjusted OR	95% Confidence Limits
VAS scores	0.0070*	1.429	1.102 – 1.852
Maximal Interincisal Distance	0.0268*	0.937	0.885 – 0.993
Gender (women)	0.0270*	2.810	1.125 – 7.022

* Statistically significant

**Figure 1:**
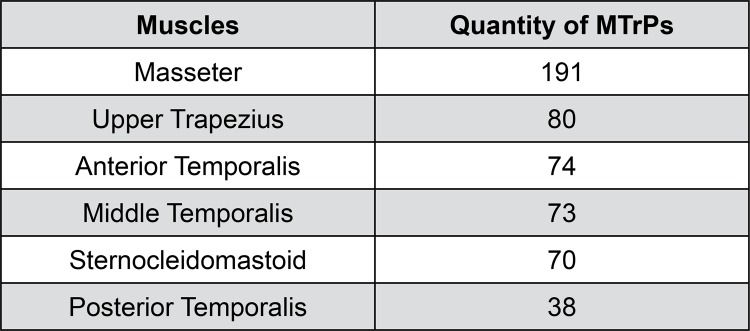
Distribution of MTrPs on the muscles evaluated, in decreasing order of prevalence

Patients in Group 1 presented 526 MTrPs, of which 491 classified as latent and 35 as active. The distribution of MTrPs on the muscles analyzed, in decreasing order of prevalence, is shown in Figure 1.

MTrPs: myofascial trigger points

## Discussion

To the best of our knowledge, this is the first study to assess the impact of MTrPs in patients with DDWR. By comparing patients with and without MTrPs, the variables VAS pain score, MID and gender (women), presented statistically significant differences*,* and were shown to be associated with the presence of MTrPs in individuals with DDWR, supporting the rejection of the null hypothesis.

TMJ-related pain is considered the most common reason for referral to a TMD specialist^1^. Overall, mean VAS pain scores found in this study (5.73±1.84) is similar to that found in a previous study with DDWR patients (6:26±1:51)^18^. When the groups were compared, VAS pain scores presented a statistically significant difference (p=0.007), and the adjusted OR demonstrated that for each increase in the VAS pain scale, the chance of a patient with DDWR to also present MTrPs increased 1:429 times. The higher VAS pain scores found in this study in patients presenting DDWR and MTrPs may be explained by the pain mechanism of each individual condition (DDWR and MTrPs), as well as the peripheral and central sensitization.

In TMJ arthralgia, peripheral neurogenic inflammation increases excitability of afferent nerve endings, resulting in peripheral sensitization of trigeminal neurons^20^. Inflammatory mediators can also spread through the peripheral tissues and increase excitability of adjacent nociceptive nerve endings^21^. The intense afferent stimulation of the central nervous system can lead to central sensitization, contributing to chronic pain, with the consequent secondary muscle involvement^21^. The main components responsible for the pain sensation of MFP are the MTrPs^12^. In MTrPs there is an abnormal depolarization of the motor end-plates of the muscle, followed by prolonged muscle contraction associated with autonomic and sensory reflex arcs supported by central sensitization^13^ ^,^ ^22^. Both active and latent MTrPs can be involved in pain sensitization processes involving the central nervous system^22^. Thus, there is a combination of sensitivity to pain and painful events, which can be self-sustained by peripheral and central sensitization phenomena^21^.

In healthy individuals, MID ranges between 45 and 53 mm^1^. In this study, mean MID of the whole sample was 42.34±8.28 mm, slightly higher than previous results obtained in patients with DDWR (39.07±4:54 mm)^18^. Comparing the groups, patients of Group 1 showed significantly smaller MID (p=0.0268), but mean next to normality (41.54±8.38). These results can be explained by the fact that, during examination, participating patients were asked to open their mouth slowly and steadily as much as possible, even in the presence of discomfort, which allowed the patient to partly overcome muscle-induced restrictions^23^. Moreover, MTrPs cover a limited area, in which only a few motor units are contracted and there is no shortening of the whole muscle^12^ ^-^ ^14^. Thereby, for every millimetre MID increased, the chance of a patient with DDWR to also present MTrPs decreased 0.937 times.

The women:men ratio in the present sample (2.4:1) is similar to the gender distribution found in previous studies (2.2:1 to 4.6:1)^5^ ^,^ ^24^. This prevalence of women was expected, as they tend to seek treatment seven times more often than men^1^. Besides that, it is believed that estrogen can increase inflammatory hyperalgesia in the TMJ, and have peripheral and central action in the modulation of pain, influencing the sensitization of the trigeminal system^25^. Among the internal disorders of the TMJ, the greater occurrence of DDWR in women^26^ may derive from the influence of some female-specific characteristics such as greater joint laxity^27^ and greater intra-articular pressure^28^. Differences in muscles are also important. Type I fibers are more prevalent in skeletal muscles of women than in men, which could lead to greater muscle sensitivity^29^. Thus, the fact that the female gender would be correlated with the presence of MTrPs in patients with DDWR was not surprising. In fact, the present results suggest that women with DDWR were 2.8 times more likely to also present MTrPs than men with DDWR.

The mean age of participants in the present sample (36.45±14.06 years) was within the range previously reported for TMD patients (20 to 40 years)^8^ ^,^ ^26^. The present results showed that age was not statistically correlated with the presence of MTrPs in DDWR patients. Previous studies have demonstrated that age is an important factor for the increased presence of pain^8^ ^,^ ^9^ ^,^ ^26^. However, in those studies, age was categorized and the sample was segmented into different age groups, which allowed for assessment of the progressive impact of pain along the years.

Although the duration of the complaint is not the only determinant factor for pain chronicity, musculoskeletal pain may be considered to be chronic when lasting for ≥6 months^1^. Mean pain duration reported in this study (31.58±46.94 months) is lower than those reported previously in the literature (58.60±59.89 months)^24^. This difference can be explained by the fact that, in the epidemiological study in question, all TMD conditions, and not only DDWR, were considered. The pain duration was not statistically different between the two groups studied, and this variable could not be correlated with the presence of MTrPs in DDWR patients.

While subjective, when muscle palpation is performed by an examiner with large experience with MTrPs, their clinical identification has been shown to be reliable^16^. MTrPs have the capacity to produce referred pain when palpated, in which the pain sensation spreads from the MTrPs itself to a reference area, and this pain is usually described as deep, diffuse, with intensity ranging from mild discomfort to disabling severe pain^12^ ^,^ ^13^. The referred pain mechanism can be explained by the convergence theory, in which several first order neurons would synapse with a single second order neuron, and therefore, becoming a persistent nociceptive stimulus that could end up producing pain in areas other than the affected^11^ ^-^ ^14^. MTrPs may be active or latent, and their clinical distinction has been supported by immune histochemical studies, in which higher levels of neuroactive mediators (bradykinin, substance P and serotonin) were found in active MTrPs when compared to latent MTrPs^30^.

In this study, 526 MTrPs were identified, of which 491 were classified as latent, and 35 active. This higher frequency of latent MTrPs was expected, since they are not only prevalent in patients with musculoskeletal pain, but also in individuals without clinical complaints, consisting of one of the potential sources of motor dysfunction^11^ ^-^ ^14^. Latent MTrPs can persist for years after the apparent resolution of the problem, but predisposes the patient to acute pain surges, which can be reactivated by slight stretching of the muscle, trauma, hyperactivity, cooling, or emotional stress^11^ ^-^ ^14^. Since the genesis of MTrPs involves excessive muscle use^12^ ^,^ ^13^, a higher occurrence is expected in the most required muscles, such as those involved in mandibular and cervical mechanics (masseter and temporalis). The high prevalence of MTrPs and the impact on VAS and MID scores in patients with DDWR found in this study indicate that, perhaps clinically, the treatment in these patients should begin with the inactivation of MTrPs. Future studies may contribute to clarify this question.

Even though the goodness-of-fit test indicated that the statistical model and the variables used were significantly well fitted (p=0.5962), there is the limitation of this study being a monocentric study, with restricted population. Although all participants presented painful complaints, no minimum number of pain level was stipulated and no mapping of referred pain pattern elicited by MTrPs palpation was conducted, and, as a result, the identification of whether joint pain symptoms were secondary to some point was not possible. Besides that, the patients’ mental health was not assessed. More investigations with the inclusion of a TMD-free control group, and/or with other specific conditions such as disc displacement without reduction, using more objective instruments of analysis, such as a pressure pain threshold algometer, as well as assessing the patients’ mental health, are suggested. In addition to that, ideally, prospective studies should be conducted in order to elucidate the impact of a particular disorder on another.

## Conclusions

In view of the results and limitations of this study, it can be concluded that patients with DDWR and MTrPs had increased VAS pain scores and a decrease of MID compared to patients with DDWR and no MTrPs. The variables VAS pain scores, MID, and gender (women) showed a significant association with the concomitant presence of DDWR and MTrPs.
